# A Rare Occurrence of Opportunistic Infection by Streptococcus mitis Due to Antibiotic-Induced Neutropenia in Prosthetic Joint Infection

**DOI:** 10.7759/cureus.35583

**Published:** 2023-02-28

**Authors:** Eng Kee Tan, Khairil Anwar Ahmad Hanif, Samuel John Jebasingam Issace, Fahrudin Che-Hamzah

**Affiliations:** 1 Orthopaedics and Traumatology, Ministry of Health Malaysia, Selangor, MYS; 2 Orthopaedics and Traumatology, Hospital Pengajar Universiti Putra Malaysia, Serdang, MYS

**Keywords:** streptococcus mitis, viridans streptococci, prosthetic joint infection, neutropenia, cefazolin

## Abstract

Prosthetic joint infection (PJI) is a devastating complication in arthroplasty surgery. Although the prevalence is less than 2%, its functional and financial implications are significant. Part of its treatment involves the usage of prolonged and high-dose systemic antibiotics. Ironically, this predisposes the patient to unwanted adverse effects caused by the drugs. We report a case of cefazolin-induced neutropenia that led to *Streptococcus mitis* (*S. mitis*) bacteraemia in a patient with *Staphylococcus aureus* PJI. There have been no previous reports on cefazolin-induced neutropenic bacteraemia complicating the treatment of PJI. This case report aims to create awareness among the attending physicians on the possibility of cefazolin-induced neutropenia, which led to bacteraemia from an opportunistic microorganism. The reversal was as simple as cessation of the antibiotic itself. However, if not recognized, it could be fatal.

## Introduction

Prosthetic joint infections (PJI) are an uncommon yet devastating complication in arthroplasty surgery. The American Academy of Orthopaedic Surgeons has found the prevalence of PJI to be approximately 1.63% following hip replacements, and 1.55% following knee replacements. Despite the relatively low prevalence, PJI causes significant morbidity to the patient and places a high financial burden on the healthcare system [1]. The main mechanisms of pathogenesis include direct seeding or inoculation during surgery, haematogenous spread, and recurrent infections [2]. As with all implant-related infections, treatment proves to be challenging and complicated. It is made worse by poor antibiotic penetration into the affected area. This leads to a prolonged administration of high-dose systemic antibiotics, despite its inherent risks of adverse effects. In patients with methicillin-sensitive *Staphylococcus aureus* (MSSA), one of the antibiotic choices is intravenous (IV) cefazolin. Although neutropenia is a known adverse effect of cefazolin, there is no reported incident of neutropenic bacteraemia during the usage of cefazolin in the treatment of PJI. We report an occurrence of cefazolin-induced neutropenia leading to an opportunistic infection by *Streptococcus mitis* bacteraemia in a patient who was diagnosed with *Staphylococcus aureus* PJI.

## Case presentation

**Figure 1:**
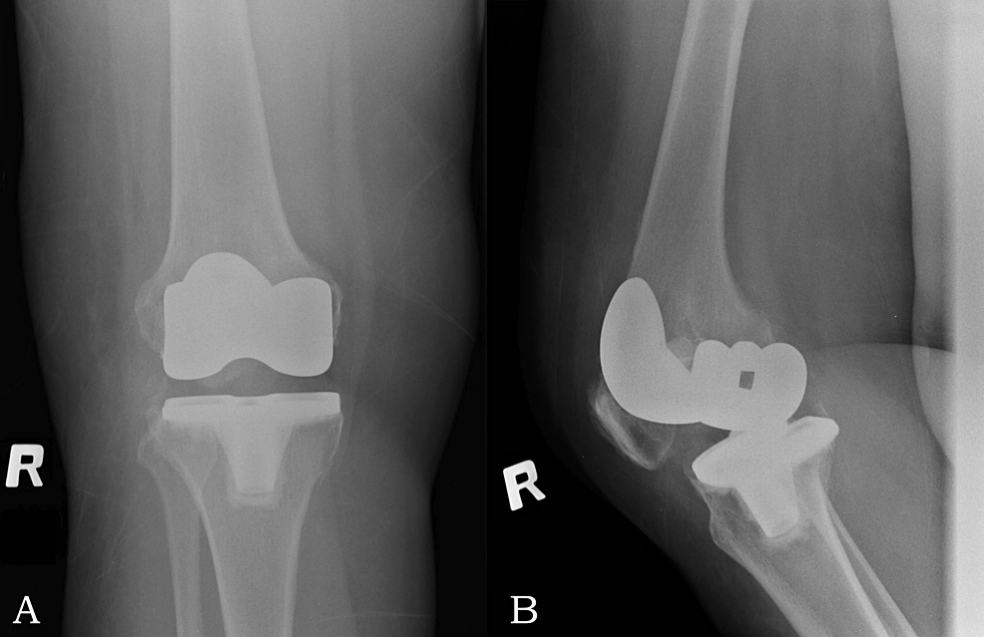
Plain radiographs of the right knee Anteroposterior view (A) and lateral view (B) showing no evidence of implant loosening.

A 71-year-old female with a history of right total knee replacement (TKR) done three years ago presented with a complaint of acute onset right knee pain with swelling for about three weeks duration. Physical examination revealed a swollen and tender right knee. The range of motion was reduced to 0-45 degrees. She remained afebrile. Plain radiographs (Figure 1) showed no evidence of implant loosening or changes indicating osteomyelitis.

**Figure 2:**
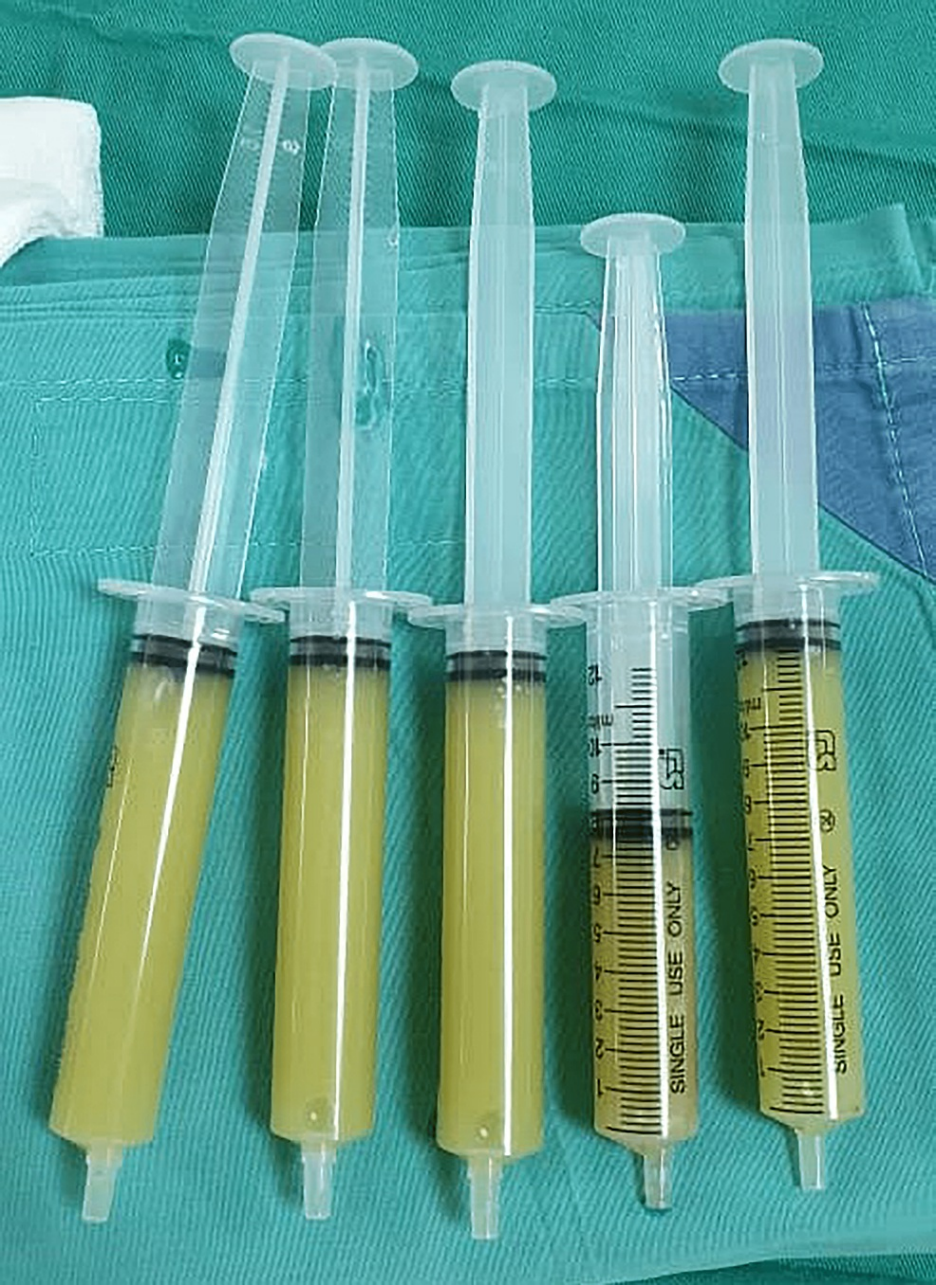
Purulent synovial fluid aspirated from the right knee joint

Her total white cell count was slightly elevated (11.4 x 10^9^ cells/microL) with an absolute neutrophil count (ANC) of 8.54 x 10^9^ cells/microL. The erythrocyte sedimentation rate (ESR) and C-reactive protein (CRP) were markedly elevated at 98 mm/hr and 256.2 mg/L, respectively. The blood culture grew no microorganisms. Right knee aspiration yielded approximately 50 ml of purulent synovial fluid (Figure 2).

Subsequently, she underwent surgical debridement, irrigation, and exchange of polyethylene insert. The femoral and tibial components were well fixed and, therefore, retained. Histopathological examination of intra-articular soft tissue showed an acute inflammatory process with granulation tissue formation. Intraoperative synovial fluid cultures revealed MSSA (Table 1).

**Table 1:** Synovial fluid culture and sensitivity

Organism: *Staphylococcus aureus*	
Antimicrobial agent	Sensitivity
Oxacillin	Sensitive
Erythromycin	Sensitive
Trimethoprim/sulfamethoxazole	Sensitive
Penicillin G	Resistant

After discussion with the infectious diseases physician, the patient was started on IV cefazolin 2 g eight hourly. Her clinical condition as well as the ESR and CRP trends were monitored closely during the course of antibiotic therapy. On day 24 of IV cefazolin, she developed two temperature spikes. At the time, her right knee was asymptomatic, with an absence of clinical signs indicating a recurred infection. There was a significant drop in ANC to 0.76 x 10^9^ cells/microL. *S. mitis* was isolated from the blood culture. The patient had developed neutropenic bacteraemia. It was postulated that neutropenia was an adverse effect of the IV cefazolin. Subsequently, the IV cefazolin was discontinued, and based on the latest blood culture sensitivity, IV ceftriaxone 1 g 12 hourly was administered for three weeks. The ANC level was monitored regularly and it normalized (2.53 x 10^9^ cells/microL) on day seven after discontinuation of IV cefazolin. Repeated blood culture grew no microorganisms. Upon discharge, the ANC level was 3.23 x 10^9^ cells/microL. The ANC trend is summarized in Table 2 below. She was discharged with an oral combination of ciprofloxacin and sulfamethoxazole/trimethoprim for three months. The patient remained infection free during follow-ups of more than three years.

**Table 2:** Summary of TWC and ANC according to days of antibiotics TWC: total white cell count; ANC: absolute neutrophil count.

Antibiotic agent	TWC (x10^9^ cells/microL)	ANC (x10^9^ cells/microL)
Cefazolin day 1	11.4	8.54
Cefazolin day 7	11.3	8.0
Cefazolin day 14	4.6	2.44
Cefazolin day 24	2.3	0.76
Cefazolin stopped and changed to ceftriaxone		
Ceftriaxone day 7	4.5	2.53
Ceftriaxone day 14	5.2	3.01
Ceftriaxone day 21	5.4	3.23

## Discussion

Treatment of PJI is complicated and should involve a multidisciplinary approach involving the orthopaedic surgeon and the infectious diseases physician. In treating patients with PJI, surgical management is used in combination with antibiotic therapy. However, various surgical methods exist, yet there have been no randomized trials to compare the outcome of these methods, as it would be difficult to conduct and perform such a study [3]. Usage of antibiotics tends to be in high doses and for a prolonged duration. Unfortunately, this puts the patients at risk of adverse effects from the administered antibiotics.

When selecting an antibiotic agent, it is important for the clinician to know and understand its effectiveness and the possible adverse events that can occur. Numerous studies have demonstrated the efficacy of cefazolin versus anti-staphylococcal penicillins (ASP). A systematic review by Weis et al. found that cefazolin was associated with lower 30-day mortality rates as well as less nephrotoxicity, in addition to being lower in cost [4]. The selection of antibiotics should also include its rate of penetration into bone and joints. In PJI, this is undeniably an important factor to consider. Thabit et al. evaluated the antibiotic penetration into bone and joints, reviewing more than 30 different antibiotics and analysing their pharmacokinetics. They found that a cefazolin serum concentration of 170.3 + 51.3 µg/ml translated to a cancellous bone concentration of 30.4 + 19.8 µg/ml in the knee. The concentration of cefazolin in synovial fluid was also equal or higher compared to serum. This exceeded the minimum inhibitory concentration (MIC) or MIC90 of *Staphylococcus* [5]. ASP had a poorer penetration into bone according to their results as most ASP, such as cloxacillin, are highly protein-bound, affecting their penetration ability. This supports the findings by Cunha et al., in their comparison between cefazolin and cephradine, where the former had higher efficacy in synovial fluid [6].

Despite its effectiveness, cefazolin is not without risks. The most common adverse effects are usually mild, such as rash, fever, and transient elevation of serum transaminase and alkaline phosphatase. An extremely uncommon yet potentially fatal complication is neutropenia. In a comparative analysis by Turner et al., patients on 14 or more consecutive days of antibiotics were assessed for neutropenia. Of the six antibiotics included in the study, cefazolin had the lowest risk of neutropenia. The onset of neutropenia was found to occur after the second week of consecutive administration [7]. In our case, neutropenia occurred on day 24 of IV administration. Following cessation of the antibiotic, the ANC improved the following week.

Neutropenia in itself poses a risk of opportunistic infections. In this case, the patient suffered from neutropenic bacteraemia caused by *S. mitis*. In a healthy individual, this organism presents as normal oral commensal flora. However, it poses a significant threat in immunocompromised patients such as those with neutropenia or patients undergoing cytotoxic chemotherapy [8]. This has led to the recognition of *S. mitis* as the leading cause of infective endocarditis and bacteraemia amongst oral streptococci in susceptible patients. The pathophysiology of how *S. mitis* causes a wide array of diseases ranging from infective endocarditis to bacteraemia and meningitis remains unknown, though it is postulated to be related to its ability to bind directly to platelets, implicating its role in the pathogenesis of diseases such as infective endocarditis. The virulence and severity of *S. mitis* infections are confirmed in a study by Shelburne et al., where *S. mitis* was found to cause the highest frequency of bacteraemia and severe disease. Multiple strains were identified, all of which were capable of causing a near-death status in mice models [9].

## Conclusions

Treating PJI remains a challenge. Currently, antibiotic therapy is the treatment for this condition. As clinicians, we should be aware of the possible adverse effects that can occur when an antibiotic is given at a higher dosage and for an extended duration. One rare occurrence of these adverse effects is neutropenia, which in turn causes bacteraemia from an opportunistic infection. When recognized, the treatment is as simple as cessation of the antibiotic itself. However, if it is not identified early, it could be potentially fatal.
